# Creating a Single Application and Approval Process to Enable Research; an example using CPRD Primary Care Data and Public Health England Cancer Registry Data

**DOI:** 10.23889/ijpds.v1i1.332

**Published:** 2017-04-19

**Authors:** Sophia Amjad, Rachael Williams, Rachael Brannan, Tariq Malik, Janet Valentine

**Affiliations:** 1 Clinical Practice Research Datalink (CPRD); 2 Public Health England (PHE)

## Objectives

To enhance the research value and capability of its primary care database, the Clinical Practice Research Datalink (CPRD) has collaborated with Public Health England (PHE)’s National Cancer Registration and Analysis Service to facilitate access to linked cancer registration data for use in research, pharmacovigilance, drug monitoring and health outcomes analysis. Since 2009, access to this linked resource has been co-managed by CPRD and PHE, through two parallel, independent approvals processes: (a) the MHRA Independent Scientific Advisory Committee (ISAC) and (b) the PHE Office for Data Release (ODR). In upholding the Office for Life Science Ministerial Industry Strategy Group (Health Data Programme)'s vision to minimise process barriers to accessing real world data, CPRD and PHE have worked together to unify and streamline these two processes into a single end-to-end application and approval process. 

## Approach 

Each organisation reviewed each other's approval processes to achieve an improved mutual understanding of the respective organisation's governance approach, the risk based assessments applied to disclosure risk, risk appetites and policies, with the goal to harmonise these into a single approval process.

## Results

CPRD and PHE are finalising a contract establishing a clear operating framework allowing CPRD to grant approval to researchers for the use of linked cancer registry data. The contract names CPRD as a joint data controller and sets out the purposes for processing, the manner of processing and the means by which joint data controller responsibilities will be satisfied. An associated service level agreement is in discussion which will enable robust timelines and performance management for both organisations. These developments are important milestones towards achieving the single approval process by allowing CPRD to review applications for cancer registry data in-house, simultaneously to the ISAC review. 

## Conclusion

The strong relationship built between CPRD and PHE, and willingness to develop a single application and approval process, will strengthen and streamline access to these data, whilst assuring patients and the public that scientific integrity is maintained and proportionate information governance checks are in place. Upon completion of this work, applicants will experience associated faster review and feedback time, ultimately leading to faster approvals. Researchers wishing to utilise these linked data will soon be able to submit one application to ISAC, have one point of contact and one approval.

